# A Retrospective Study of Deep Vein Insufficiency Treatment Device: ICT

**DOI:** 10.3400/avd.oa.20-00016

**Published:** 2020-09-25

**Authors:** Turhan Yavuz, Altay Nihat Acar, Kubra Yavuz, Evren Ekingen

**Affiliations:** 1 Department of Cardiovascular Surgery, Süleyman Demirel University Faculty of Medicine; 2 Batman Regional State Hospital

**Keywords:** deep venous insufficiency, interventional treatment

## Abstract

**Objective**: This study aimed to evaluate the efficacy and safety of a newly developed, leak closure Internal Compression Therapy (ICT) (Invamed, Ankara, Turkey) device during a single-session procedure in a group of patients with primary deep valve incompetence.

**Methods**: There were 286 patients who were diagnosed with deep venous reflux by duplex scanning. They underwent valvular leak operations to treat primary deep venous insufficiency. Follow-up visits were on the third day, first month, sixth month and twelfth month. At each visit, duplex scanning and a clinical examination were performed. Successful treatment was defined as deep vein valves without reflux. Any patency or reflux over 1 sec was considered a failure.

**Results**: The study enrolled 286 patients with deep venous insufficiency. Procedural technical success was 100%. At the one-year follow-up, the overall success, among all patients, was 92%. No significant morbidity or mortality related to the procedure were observed. All patients had major improvements in venous clinical severity score (VCSS) scores postoperatively. VCSS scores at pre-intervention, and at the twelfth month, were 20.7±5.9 and 3.9±0.9, respectively (p<0.001).

**Conclusion**: After the twelve-month follow-up, the postprocedural outcomes indicate the ICT device is safe and effective.

## Introduction

Chronic venous insufficiency is a common reason for decreased quality of life. The most severe forms are those caused primarily by post-thrombotic syndrome affecting the deep venous system^1)^ and, to a smaller scale, congenital and primary types.^2)^ Compression therapy is accepted as the primary form of treatment,^3)^ but complete control of the disease is not always possible. In a select group of patients, varied treatments can be performed for reflux treatment or re-establishing venous flow. For treating patients with valve insufficiency primary, treatment appears to be valve reconstruction.

The definition of deep venous reflux (DVR) in the lower limbs is deficiency of transferring venous blood to the caval system in the deep venous system. The reflux pattern can be either axial or segmental. The definition of a deep segmental reflux is a confined reflux in a segment of the femoral, popliteal, crural, or calf muscle vein. Axial reflux is defined as a continuous reflux in all segments of the veins from the groin to calf. This axial reflux can originate from the deep venous system or can also be present in the superficial venous system connected to the deep venous system by perforator veins. Three possible aetiologies may cause DVR. According to the clinical, etiologic, anatomic and pathophysiologic data (CEAP) classification, they are: Es (post-thrombotic syndrome), Ep (primary deep valve incapability) and Ec (congenital valve malformation). Es is the most frequent etiology (60–85% of cases) in particular series,^4)^ and Ep in other studies.^5)^ In the etiology of post-thrombotic syndrome, valve deficiency is a outcome of thrombosis and vein wall inflammation. After the recanalization stage, because the valve is partially or totally damaged, fibrosis and retraction occur. In post-thrombotic syndrome (PTS), valve repair is not considered feasible.^6)^ The secondary cause of DVR is primary deep valve incompetence (PDVI). The anomalies that are observed most frequently are the long and abnormal free edge of each valve, valve ring dilatation with a widening of the commissures, asymmetrical leaflets, or cusp insertion. These anomalies can usually be fixed by valvuloplasty.^7,8)^

**Figure 1:**
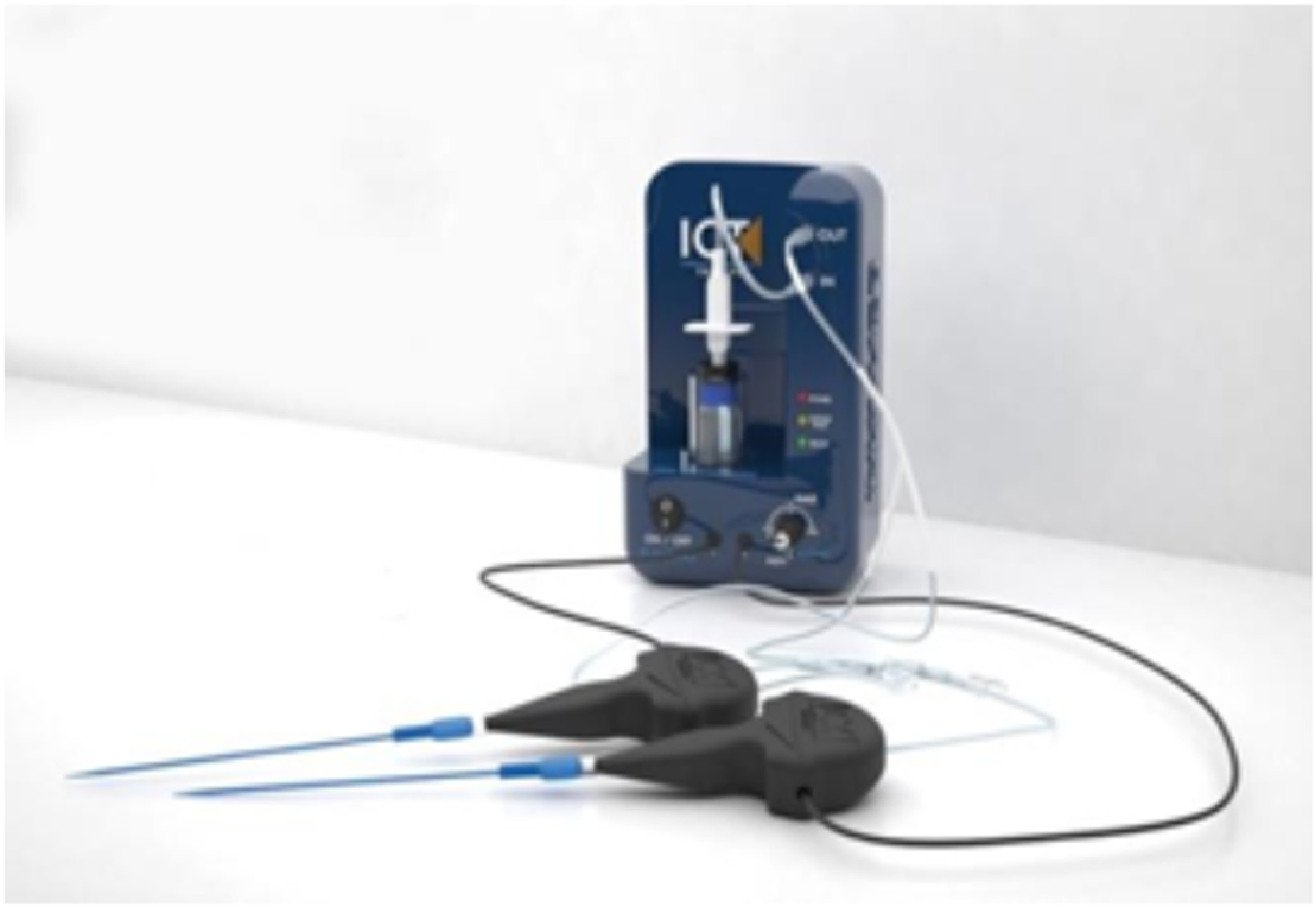
Fig. 1 Internal Compression Therapy Paravalvular Leak Closure Device and Delivery System, Percutaneous Exovenous Reconstruction System.

A newly developed technology offers a percutaneous interventional approach to PDVI for the first time. The Internal Compression Therapy (ICT) Paravalvular Leak Device (Invamed, Ankara, Turkey) is an injection pump operated implant device, which injects a combination of hyaluronic acid and n-butyl-cyanoacrylate (n-BCA) hard gel implant over the insufficient deep vein valves between the deep vein and muscle fascia (**Fig. 1**). The aim is to close the gap between the vein valves with the hard gel implant. The implant sticks and stays over the valves. It can move with muscle pump mechanism and helps insufficient valves to operate functionally.^9)^ The purpose of this study was to evaluate the efficacy and safety of the ICT device during a single-session procedure in a group of patients with PDVI.

## Materials and Methods

Patient selection. Between March 2017 and September 2018, 286 valvular leak operations were performed in 286 patients (172 male, 114 female, median age 55, range, 36–80) affected by primary deep venous insufficiency. All venous insufficiencies were resulted from abnormal coaptation of leaflets. Patient demographics are shown in **Table 1**. All patients were classified according to CEAP and Venous Clinical Severity Score (VCSS) classification.

**Table 1:** Table 1 Demographics

	Mean±Std (n)	N (%)
Age (years)	55.0±13.2	
Gender (male–female)		172 (60.2%)–114 (39.8%)
Diameter (mm)	12.4±2.6	
Distance between cusps of the valve (mm)	3.9±1.3	
Reflux (sec)	3.6±1.0	
CEAP category		
C3		114 (39.8%)
C4		104 (36.3%)
C5		36 (12.5%)
C6		32 (11.1%)
VCSS	20.7±5.9	

CEAP: clinical, etiology, anatomy and pathophysiology classification; VCSS: venous clinical severity score

An animal model of this treatment was carried out at the Cukurova University Experimental Research Center with approval of the Cukurova University Local Ethics Committee for Animal Experiments. EC Design—Examination Certificate and Full Quality Assurance System Certificates were obtained. The ethics committee of the Suleyman Demirel University Clinical Research Ethics Board of Medical Faculty approved the study (No. 72867572-050-11446).

This sample of patients was selected from among 345 patients. After patients were confirmed to be eligible and provided written informed consent, a clinical examination was performed by a senior surgeon, and duplex scanning was performed by an independent radiologist.

All patients underwent duplex scanning with complete mapping of the perforator deep and superficial venous system. Ultrasound scanning was performed in the standing position using a standard technique. CEAP, VCSS assessments, and duplex scanning (DUSG) results were documented.

### Selection criteria

All patients presented with a DVR assessed by DUSG. The patients, who were diagnosed with primary deep vein incompetence were selected by DUSG. Patients with absent valves, fixed (thickened) valves, mono-cusp and other destructive valves were excluded. The CEAP classification score was C3–6, Ep, Ad, Pr among patients. Patients with ulcer secondary to deep venous insufficiency, presented with an untreatable ulcer despite conservative therapy and perforator and/or superficial treatments. The ulcer was present for at least one-year or recurred more than once. The ulcer was a reason for the patients’ major discomfort and pain.

Exclusion criteria were:
  ·Severely limited ambulation
  ·Thrombophilic syndrome
  ·Post-thrombotic etiology
  ·History of deep vein thrombosis
  ·Anticoagulant usage contraindications
  ·Severe comorbidity
  ·Feasibility of standard techniques (femoral transposition,^10)^ valve transplant^11,12)^)
  ·DVR <2 sec

### Procedure

**Figure 2:**
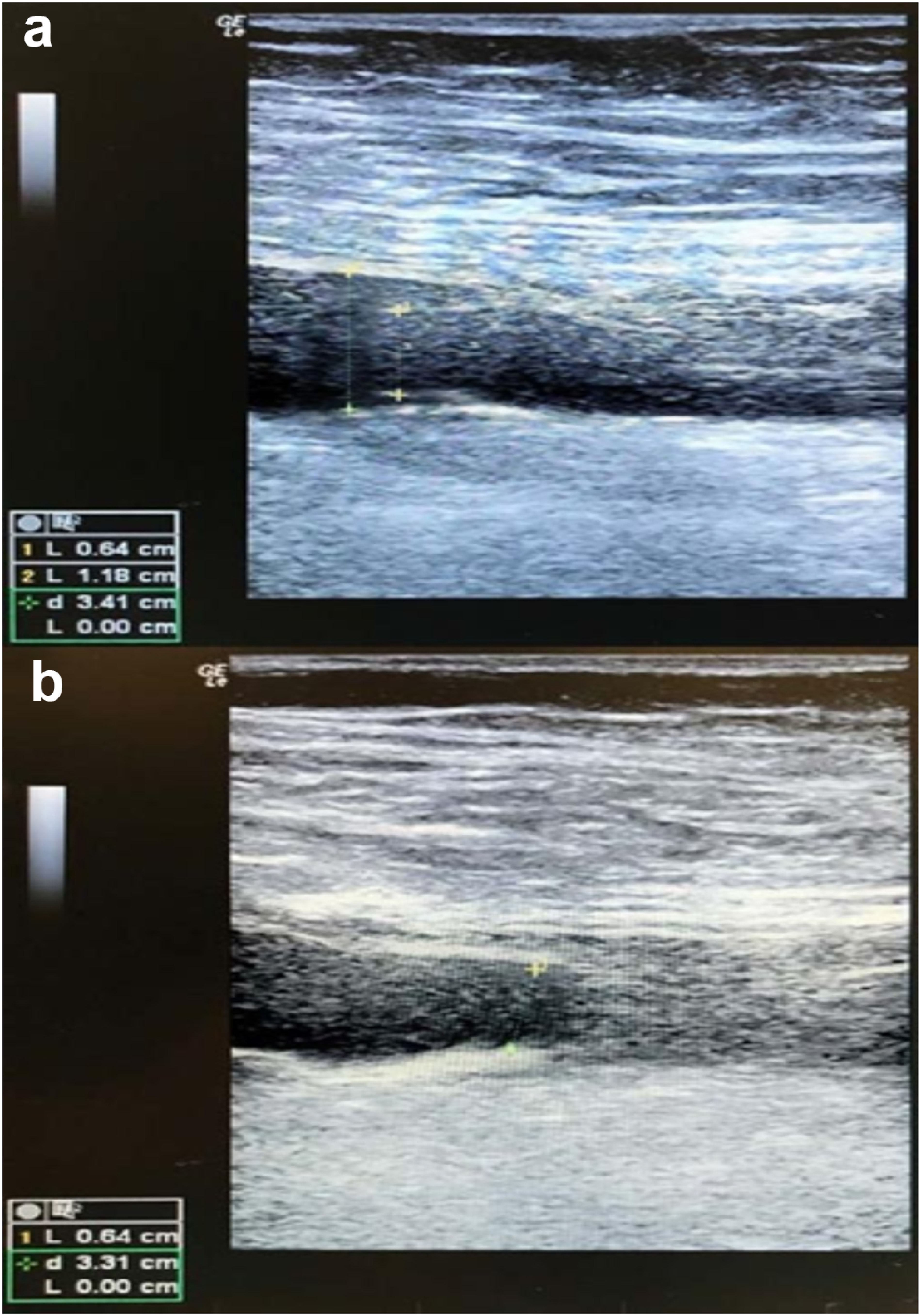
Fig. 2 (**a**) Measurement of the gap between insufficient valves and vein diameter under ultrasound; (**b**) measurement of the vein diameter and reflux control after the procedure.

All procedures were performed by using a standard sterile technique under local anesthesia. The patients were placed on the operating table in the supine position, with the knee flexed to 90 degrees. The treatment goal was to decrease the vein diameter until the gap between valve cusps closed. The most proximal insufficient deep vein valve position was confirmed with ultrasound. The gap between insufficient valve cusps and the vein diameter were measured (**Fig. 2a**) . The ICT device consists of leak closure device with a controlled mix unit, injection unit, and two vials of hyaluronic acid polidocanol, Ethylene–Vinyl Alcohol Copolymer (Libro) and a very small amount of n-BCA and delivery system with aspiration and injection connector, delivery line and 2 units of 6F, 11 cm cannulas (**Fig. 1**).

**Figure 3:**
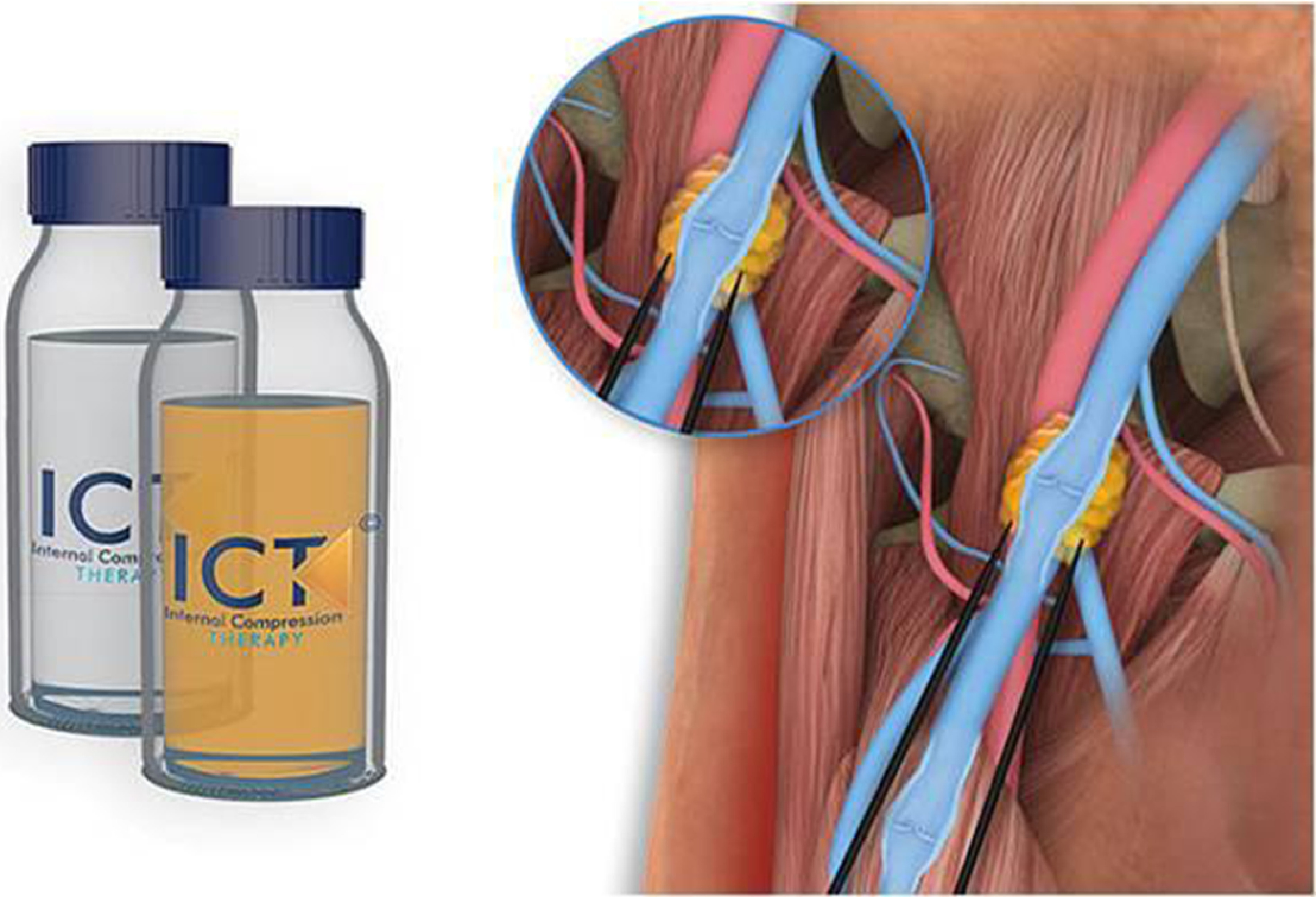
Fig. 3 Delivery system cannula placement over to the two sides of the vein valve.

The most proximal valve area was selected for the procedure to prevent proximal reflux. Puncture needle entry was performed on the area between muscle fascia and deep vein. Puncture needle entry was performed twice around the opposite deep vein in order to inject the hard gel of ICT equally around the deep vein. After puncture needle entry achieved 0.035, a 45 cm guidewire was inserted and movement of the guidewire over the deep vein was observed. The puncture needles were withdrawn and 6F, 11 cm delivery system cannulas advanced over the wire to the two sides of the vein valve (**Fig. 3**).

The hard gel of ICT device comes in two different 2 ml vials as hyaluronic acid and n-BCA. Before injection over the valves, hyaluronic acid and n-BCA were mixed with the ICT device mix unit for 30 sec with a prefixed program. The device’s aspiration and injection ports were connected to the mixed vial and cannulas, respectively. The ICT device’s injection unit was activated and adjusted to the desired injection speed. Using ultrasound, injection began and the vein diameter decreased to the difference between the vein diameter and the gap between valve cusps. If an excess amount of hard gel were injected, the device’s suction unit reversed, and aspiration was performed immediately. After the gaps between the valve cusps were completed, the function of the valve and reflux were controlled (**Fig. 2b**). The injection step of the procedure was performed again in case of existing reflux. After confirmation of reflux again, the cannulas were retracted and compression stockings (20–30 mmHg) were used and prescribed for long term use.

### Follow-up

Follow-up visits were performed at the third day, first month, sixth month, and twelfth month. At each visit, an independent ultrasound study and a clinical examination were performed. Treatment success was defined as deep vein valves without reflux. Any patency or reflux over 1 sec was considered a failure.

### Statistical analysis

Complete elimination of reflux of the deep vein was calculated using Kaplan–Meier methods. A paired t-test was performed to determine changes in VCSS baseline between control periods. Values are expressed as number and percentage (n, %) or mean±standard deviation. All comparisons were made using the SPSS v.22 statistically.

## Results

In the study, 286 patients aged 36 to 80 years with deep venous insufficiency were enrolled. No patients were lost to follow-up. Patients (114 women [39.8%] 172 men [60.2%]) were a mean age of 55.0±13.2.

By the CEAP classification, 114 patients (39.8%) were C3, 104 (36.3%) were C4, 36 (12.5%) C5 and 32 (11.1%) were C6. The average preprocedural VCSS was 20.7±5.9 (range 11–30). The mean preprocedural diameter of the deep vein at the valve level in the standing position was 12.4±2.6 mm (range 9.0–17.2), distance between valves was 3.9±1.3 (range 2.0–6.5) with a mean reflux of 3.6±1.0 s (range 2–5). The average ICT hard gel delivered was 2.3±0.6 ml (range 1.8–3.1). The mean procedure time was 11.3±5.2 min (range 6–16).

Procedural success was 100%. During one-year follow-up, overall success among all patients was 92%. No significant morbidity or mortality related to the procedure was observed. No ecchymosis, skin pigmentation, hematoma, paresthesia, deep vein thrombosis, or pulmonary embolism was observed. Complete ulcer healing observed in 78% of the patients with median period of 10 weeks (range 3–18). The deep vein diameter after procedure was 9.0±1.7 mm. All patients had significant improvement in VCSS scores postoperatively. The VCSS scores at pre-intervention and at the twelfth month were 20.7±5.9 (range 11–30) and 3.9±0.9 (range 3–5), respectively (p<0.001) (**Table 2**).

**Table 2:** Table 2 Clinical Assesment

VCSS	Mean±Std (n)	P value
Pre-Op	20.7±5.9 (286)	
First month	7.0±2.5 (286)	p<0.001
Sixth month	5.3±1.4 (286)	p<0.001
Twelfth month	3.9±0.9 (286)	p<0.001

VCSS: venous clinical severity score

## Discussion

This study is the first to analyze the mid-term clinical results of a new paravalvular leak device system in PDVI patients. Results from this study verify that ICT system is feasible and highly beneficial for treating deep venous insufficiency. No serious adverse effects were encountered during the 12-month follow-up. Until now, no toxicological, carcinogenic, or mutagenic effect has been reported for either hyaluronic acid or n-BCA over vascular or in vascular use.^13–16)^

Corrective surgery in DVR is not practiced often. The reasons are unclear; many consider it risky, while others consider it ineffective. Actually, this type of operation is not aggressive and has a low complication rate. However, in terms of efficacy, surgical procedures are still debatable and there is no convenient treatment for deep venous insufficiency.^17–19)^

Ragg et al. used hyaluronan (hyaluronic acid) in a superficial venous insufficiency, combined with schlerotherapy, in order to compress the vein. Hyaluronan compression was successful for reducing the vein diameter. Clinical investigations up to 8 weeks did not reveal any symptoms, visible inflammation, or staining in segments covered with hyaluronan. Perivenous hyaluronan did not induce any discomfort or side effects during follow-up.^15)^

Several studies showed n-BCA is safe and effective for intra vascular use in superficial venous insufficiency.^20–24)^ n-BCA sticks to the vein walls and, with pressure, vein walls stick together. Combined with hyaluronic acid, n-BCA has an attachment effect and helps hyaluronic acid to stay over the targeted vein segment.

When injecting the ICT hard gel, we attempted to obtain a complete circumferential distribution by injecting over two sides of the vein and allow the gel to distribute completely. The presented technique allows making a permanent exo-skeleton over the vein that can move with muscle fascia and provide necessary muscle movements for the first time in a new quality: Invisible, unperceived by the patients and not requiring any further care (unlike textile compression media), and with no limitations affecting work, sport, or hygiene. The exact volume required for vein compression while avoiding inflammatory symptoms is unknown; however, the target used here reduces the distance between valves in diameter proved to be a good estimate, which turned out to be effective, but imperceptible, by the patient.

Interventional DVR treatment techniques are still experimental, and treatment of subinguinal refluxes still presents various problems. In such instances, surgery is the only option at our disposal for the time being, but the indications are still questionable,^25)^ especially in post-thrombotic syndrome, Reflux treatment surgeries are not always beneficial. The search for alternative techniques^26–29)^ is what led us to developing new treatment methods. The mid-term results are encouraging, and it would be favorable to inquire into other elements, such as the most proper reconstruction site or the possibility of establishing anti-reflux mechanisms at various levels, such as the great saphenous vein, anterior accessory saphenous vein, and the small saphenous vein, so long as the minimum depth of 10 mm below the skin is respected.

Although this is a single center retrospective study with a newly developed technique, it has several limitations. Considering just the clinical routine was followed, clinical follow-ups may not determine patients’ manifestations. Given that our focus was primarily on the technique and deep vein insufficiency and reflux, investigation of collateral factors for instance parallel refluxes which are considered to be the cause of early and late failure. Additionally, the overall cost of treatment was not investigated. Because this is a simple procedure under local anesthesia, early recovery is likely.

## Conclusion

After the twelve-month follow-up, postprocedural outcomes are safe, applicable, and sufficient. With this technique, the great majority of PDVIs can be treated. This study indicates that an initial and permanent reduction in deep vein diameter, and it can be acquired by injection of a gel implant as exo-skeleton and prevent valvular leak. The lack of post-interventional symptoms is a favorable outcome. The method offers the possibility for invisible, comfortable, permanent vein compression. Initial findings are beneficial; however, long term results and randomized trials are required to confirm these results.
